# Editorial: Movement behaviors (sleep, sedentary behavior and physical activity) and physical and mental/cognitive health

**DOI:** 10.3389/fpsyt.2023.1252986

**Published:** 2023-08-07

**Authors:** Eduarda Sousa-Sá, Caylee Cook, Jade Burley, Rute Santos

**Affiliations:** ^1^Centre for Research in Sport, Physical Education, Exercise and Health (CIDEFES), Universidade Lusófona, Lisbon, Portugal; ^2^Research Centre in Physical Activity, Health and Leisure, Faculty of Sport, University of Porto, Porto, Portugal; ^3^Laboratory for Integrative and Translational Research in Population Health (ITR), Porto, Portugal; ^4^University of the Witwatersrand, Johannesburg, South Africa; ^5^University of Wollongong, Wollongong, NSW, Australia; ^6^Institute of Education, University of Minho, Braga, Portugal; ^7^Research Centre in Child Studies, University of Minho, Braga, Portugal

**Keywords:** sleep, sedentary behavior, physical activity, health, lifespan

Robust evidence has shown that all (non)movement behaviors (sleep, sedentary behavior, and physical activity) play an important role in children's growth and development (1–3) (physical, cognitive, and social-emotional) and, due to their tracking features, it may have significant health effects across the lifespan (4–8). Moreover, mounting evidence shows that an optimal (non)movement behavior combination plays a crucial role in one's health (9–13).

Traditionally, all (non)movement behaviors have been studied independently of each other. Research has shown that sleep is essential for physical and mental health at all ages (2, 14–16). It is during sleep that the human body goes through a plethora of physiological mechanisms, mainly cellular repair and rejuvenation processes (17); therefore, a lack of optimal sleep duration can have serious consequences (18, 19). Studies have shown associations between insufficient sleep or longer sleep duration and increased risk of obesity, diabetes, cardiovascular disease, and mental health issues such as depression and anxiety (2, 14–16); therefore the importance of making sleep a priority, while aiming to regularly attain the recommended hours of sleep per night, age-specific (20–23). Regarding sedentary behavior, studies have also associated it with negative health outcomes, across the lifespan. Research has shown that excessive sedentary behavior is associated with an increased risk of obesity, type 2 diabetes, cardiovascular disease, and some types of cancer (24–29). Additionally, prolonged sitting has also been linked to deleterious health effects (30–32), such as musculoskeletal pain (33, 34) and poor mental health (35). On the other hand, physical activity has been reported to have numerous health benefits, throughout lifespan (36). Regular physical activity has been associated with better physical and mental/emotional health. Specifically, higher levels of physical activity are associated with improved cardiovascular health (37), lower risk of having diabetes (38), obesity (39), hypertension (40), and some types of cancer (41). For mental health, regular physical activity has been shown to improve cognitive function and reduce the risk of cognitive decline, anxiety, depression, and dementia later in life (36, 42). Therefore, it is of vital importance for public health to not exceed the existing guidelines for sedentary behavior and to engage in at least age-appropriate levels of physical activity (20, 21, 43).

Recently, researchers have taken interest in how the different (non)movement behaviors make up a whole day (24-h movement behaviors); i.e., different patterns of movement combinations and how they might potentially affect health-, growth-, developmental-, and cognitive-related outcomes. Attaining the recommended levels of the three-movement behaviors throughout one's lifetime may be the cornerstone for healthier societies and sustainable healthcare services.

Therefore, it seems paramount to further increase our understanding of the co-dependence relationship between movement behaviors and their associations with growth, health, and developmental outcomes. Conversely, additional research is needed to determine the mechanisms of these complex, dynamic, and potentially synergetic relationships, which are not yet fully elucidated. This will potentially help in determining the most practical and effective lifestyle strategies, together with designing better-targeted interventions for promoting healthy populations.

As such, this Research Topic gathers important data to advance scientific knowledge, address research gaps, frame future research questions, and ultimately improve our knowledge in this field. This study published on this Research Topic addressed movement behaviors across all ages [from children aged 6 years (Chin, Yao, et al.) to adults aged 65+ years (Sung et al.)], as well as people with and without diagnosed conditions/diseases (Yu et al.; Chin, Wang, et al.; Sung et al.) or disabilities (Zahra et al.). This Research Topic also comprises studies conducted during the COVID-19 pandemic.

In apparently healthy people, the study of Chin, Yao, et al. showed that the COVID-19 lockdown significantly impacted children's and adolescents' sleep (increased total sleep time, more sleep onset delay, fewer sleep duration problems, less parasomnia, fewer sleep breathing problems, and less daytime sleepiness) and functioning (trends of increased emotion, behavior, and inattention problems, as well as significantly increased disturbance in home living). Male, younger children, and adolescents were the most affected by the lockdown. Moreover, it is also reported that adolescents exhibiting clusters of unhealthy behaviors (i.e., insufficient physical activity, increased screen-based sedentary behavior, and frequent sugar-sweetened beverage consumption) were more likely to present depressive symptoms than those who had no or only one unhealthy behavior (Bui et al.). Finally, the study by Colato et al., involving American college students, showed that knowing someone who had died of SARS-CoV-2 infection and having received a positive SARS-CoV-2 test result was associated with shorter sleep duration. These three studies highlight the importance of strengthening public health interventions to improve physical activity and decrease sedentary behavior levels, as well as to raise awareness about the importance of sleep. They also emphasize that different life experiences/periods may impact sleep differently, so further research is warranted to better understand how unusual events, such as COVID-19, potentially impact human sleep.

In people with and without diagnosed conditions/diseases or disabilities, Yu et al. have shown that, in children aged 8–13 years, the development of gray matter volume in the frontal cortex (which is known to be associated with attention), as assessed by neuroimaging, was highly sensitive to the effects of obstructive sleep apnoea, i.e., children with obstructive sleep apnoea undergo a cognitive impairment when compared to children without obstructive sleep apnoea. In young adults with narcolepsy, the results of Chin, Wang, et al. showed that only physical role functioning improved after a 5-year follow-up, indicating that narcolepsy cannot be cured by medication alone and that an exercise programme should be developed and recommended for patients with narcolepsy. Nevertheless, regarding psychological health domains, the results showed a significant improvement in the emotional role functioning and social functioning of patients with narcolepsy after medication treatment during the 5-year follow-up. As for the effects of physical activity and sedentary sitting time on the psychological quality of life of people (aged 15–55+ years) with and without disabilities, some of the results of Zahra et al. align with previous research, while others are novelty. This study confirmed that optimum levels of physical activity are strongly associated with better psychological quality of life amongst individuals without disabilities. Their novelty results showed that those of younger age and being single were significant predictors of poor psychological health amongst non-disables, while an increase in sedentary sitting time was significantly associated with poor psychological quality of life amongst both groups (with and without disabilities). Finally, a nationwide population-based study by Sung et al. found that patients with Helicobacter pylori infection were associated with an increased risk of sleep-related movement disorders, with a higher risk in men than in women, in those aged ≥65 years, and those diagnosed for more than 5 years.

The results of these four studies suggest the need for tailored public health policies to encourage physical activity and reduce sitting hours so that psychological health can be optimized, with a special focus on individuals with disabilities. Moreover, more research is necessary to explore whether *Helicobacter pylori* infection eradication can reduce the risk of developing sleep-related movement disorders.

In conclusion, (non)movement behaviors have a significant impact on our physical and mental health. To maintain optimal health, we should prioritize sleep, reduce sedentary behavior, and engage in regular physical activity. Small changes to one's daily routine can have a significant positive impact on one's overall health and wellbeing.

## Author contributions

All authors listed have made a substantial, direct, and intellectual contribution to the work and approved it for publication.
